# Entomotherapy: a study of medicinal insects of seven ethnic groups in Nagaland, North-East India

**DOI:** 10.1186/s13002-021-00444-1

**Published:** 2021-03-22

**Authors:** Lobeno Mozhui, L. N. Kakati, Victor Benno Meyer-Rochow

**Affiliations:** 1 Department of Zoology, Nagaland University, Lumami, Nagaland 798627 India; 2 Department of Ecology and Genetics, Oulu University, SF-90140 Oulu, Finland; 3 Agricultural Science and Technology Research Institute, Andong National University, Andong, 36729 Republic of Korea

**Keywords:** Entomotherapy, Fidelity level, Informant consensus factor, Medicinal insects, Traditional knowledge

## Abstract

**Background:**

The ethnic communities in Nagaland have kept a close relationship with nature since time immemorial and have traditionally used different kinds of insects and their products as folk medicine to treat a variety of human ills and diseases. The present study was conducted to record the entomotherapeutic practices of seven different ethnic groups of Nagaland.

**Method:**

Documentation is based on semi-structured questionnaires and group discussions with a total of 370 informants. The data collected were analysed using fidelity level (FL) and informant consensus factor (ICF).

**Results:**

Fifty species of medicinal insects belonging to 28 families and 11 orders were identified in connection with treatments of at least 50 human ailments, of which the most frequently cited were coughs, gastritis, rheumatoid arthritis, stomach ache and wound healing. *Mylabris* sp. showed the highest fidelity level (FL) of 100% for its therapeutic property as a dermatologic agent, while the informant consensus factor (ICF) ranged from 0.66 to 1.00. The use of medicinal insects varies amongst the seven ethnic groups, suggesting that differences in cultures and geographic location can lead to the selection of specific insect species for specific medicinal purposes. The largest number of insect species appear to be used for treating gastrointestinal, dermatological and respiratory diseases.

**Conclusion:**

The list of medicinal insect species, many of which are reported for the first time in the present study, suggests the presence of a considerable diversity of therapeutically important insect species in the region and elaborate folk medicinal knowledge of the local ethnic groups. This knowledge of insects not just as a food, but also as therapy is passed down verbally from generation to generation, but is in danger of being lost if not documented in a systematic way. Having stood the test of time, traditional folk medicinal knowledge and its contribution through entomotherapy should not be regarded as useless as it has the potential to lead to the development of novel drugs and treatment methods.

**Supplementary Information:**

The online version contains supplementary material available at 10.1186/s13002-021-00444-1.

## Introduction

Humans share the planet with a bewildering variety of animals and plants, forming an intricate web of interactions. Although plants and plant-derived materials make up the majority of the ingredients used in most traditional medical systems worldwide, whole animals (vertebrates as well as invertebrates), animal parts and animal-derived products also constitute important elements of the material medica [1]. The traditional medical knowledge as a part of local cultures has played an important role in identifying biological resources worthy of scientific and commercial exploitation [2–4]. Eggs, larvae, pupae and adults of certain insect species have been components of the human diet for thousands of years, be it as a regular food item or sustenance during famines, as an ingredient of medicines or part of ritual practices and even novelties.

The intertwining of the origin of the medicinal use of insects with their use as food is apparent from recorded history, but the use of insects purely as food to promote health cannot always be clearly separated from the insects’ and their products’ role solely to fight disease [4, 5]. It has been suggested that by the time insects were prescribed for therapeutic purposes by traditional healers and practitioners in South America, people were more familiar with the idea of eating them [3]. However, in Europe, it seems to have been the opposite with medicinal uses predating culinary uses [4, 5]. With the development of modern drugs, folk medicinal practices involving insects gradually became sidelined and dismissed, often seen as superstition or outright nonsense, because of weird and uncommon instructions how to carry out the procedures that supposedly would heal body and soul. However, some of the recommended remedies have stood the test of time and done well with some scientific validation [6–8]. Yet, overall medically important terrestrial arthropods have not yet benefitted much from the upswing in activity or the current interest in food insects and have received far less attention than the latter. Figures provided by Meyer-Rochow [5], in which Google searches for ‘*entomophagy*’ and ‘*insects as human food*’ yielded 140,000 and 10,300 hits but searches with ‘*entomotherapy*’ and ‘*medicinal insects*’ only resulted in 11,100 and 7110 respective hits, underscore this fact.

It has been reported that worldwide at least 1000 species of insects are used therapeutically and given the dearth of knowledge in this field the real figure may be considerably higher [5]. Approximately 300 medicinal insect species distributed in 70 genera, 63 families and 14 orders are reported from China alone [9] and hundreds more of insects to treat diseases of humans as well as domestic animals have been reported from many other parts of the world, to name but a few: Tibet [10], Japan [11], Korea [12–14], India [15–19], Spain [20, 21], Turkey [22], Africa [23, 24], South America [3, 25] and numerous more summarized in [5]. However, except for certain preliminary works in the field of ethnozoology [26–29] and recent work on entomophagy [30–35], a detailed study focusing on entomotherapy is lacking for North-East India’s Nagaland.

As part of the Indo-Burma region, Nagaland is one of the major biodiversity hotspots in the world [36] with the ethnic communities of the region that have kept a close relationship with nature since time immemorial. Naga people like hundreds of other ethnic communities of the world are known to use different kinds of plant and animal food products as remedies to treat their sick. However, given the dissimilarities in culture, customs and habits amongst the various Naga tribes and the geographic and climatic characteristics of the distinctive regions, differences are to be expected in regard to the appreciation of insects as food/medicine and the way specimens are gathered and processed by the tribals [26–32]. Although spiders, centipedes and myriapods are arthropods like insects and together with other invertebrates like snails and earthworms are widely used therapeutically [5], the present work focuses solely on insects, because it would have been beyond the scope of this investigation to also consider invertebrates other than insects. The aim of this research has been to record the folk traditional knowledge, regarding medicinal insects, present in seven different ethnic groups of Nagaland that the first author of this paper had an opportunity to interview and work with. To what extent other invertebrate species are used therapeutically and how Naga tribes other than those covered in this publication use invertebrates to treat illnesses as well as physical and mental disorders must remain subjects of future investigations.

## Materials and methods

### Study area

Nagaland is a state located in the north-eastern part of India covering an area of 16,579 km^2^. It is situated at 93° 20′–95° 15′ E and 25° 6′–27° 4′ N, in the confluence of East Asia, South Asia and Southeast Asia. Considered one of the biodiversity hotspots (within the Indo-Burma region) of the world, the state enjoys a unique geographical location and varied altitudinal range. Out of the total geographical area, 85.43% (14,164 km^2^) constitutes forest cover, of which 5137 km^2^ is dense and 9027 km^2^ open forest. Agriculture is the main economy of the state, which includes not only crop growing but all other allied activities such as animal rearing, i.e. poultry, horticulture, pisciculture, sericulture, silviculture, livestock, e.g. dairy cattle such as buffalo, cow, gayal (also known as mithun) as well as goats and pigs, etc. Two types of farming systems—jhum or shifting cultivation and terrace or wet cultivation—are practiced by the ethnic groups. Jhum cultivation is an extensive method of farming in which the farmers rotate land rather than crops to sustain livelihood [37]. Areas of jhum land are cleared once in five to eight years for better crop production during which farmers come into contact with a wide variety of insects. In terrace cultivation, the entire hillside is cut into terraces, irrigated by a network of water channels that flow down from one terrace to the other and easier to maintain than the jhum plots. However, due to the state’s wide altitudinal variation, terrace cultivation is found only in some rural pockets and the majority of the population are engaged in shifting cultivation. Rice (*Oryza sativa* L.) is the dominant crop and the main staple food of the Nagas, although certain cereals like maize (*Zea mays* L.), millet (*Eleusine coracana* (Gaertn.), *Setaria italica* (L.) P. Beauv., *Pennisetum typhoides* (Burm.) and Job’s tears (*Coix lacryma-jobi* L.) are also cultivated.

**Fig. 1:**
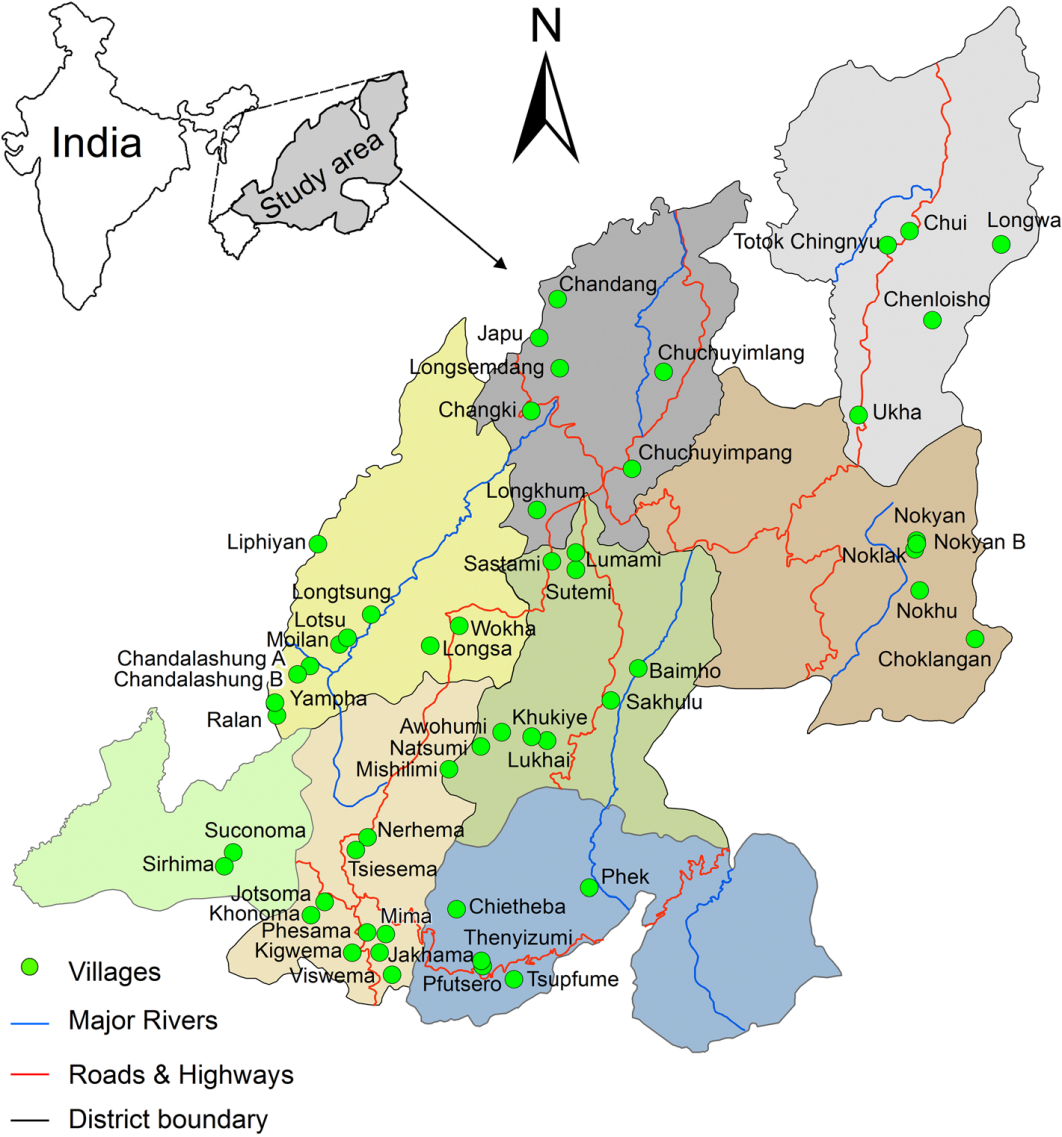
Location map of selected villages for the study (Mozhui et al. [32])

The present study is based on a 4-year field survey from 2014 to 2018 involving 53 villages (Fig. 1) across eight districts *viz*. Dimapur, Kohima, Mokokchung, Mon, Noklak, Phek, Wokha and Zunheboto in Nagaland. The target groups for the study were the Angami, Ao, Chakhesang, Khiamnuingan, Konyak, Lotha and Sumi tribes, having respective representations of 7.2%, 13.3%, 7.7%, 2.2%, 14.0 %, 8.5% and 13.9% of the total tribal population of Nagaland [38]. Members of the mentioned tribes differ from each other not just physically but speak different dialects and follow different customs and habits [26]. Demographic patterns of informants, design of semi-structured questionnaire, etc. used in the present study are available from http://www.mdpi.com/2304-8158/9/7/852/s1 [32]. Informants, who were all nominally Christians, were selected purposively on the recommendation of the community head, who was deemed the most knowledgeable and influential person. The survey was conducted only after getting ethical approval from Nagaland University, the village heads as well as the informants themselves. Therefore, with the help of semi-structured questionnaires, personal interviews with 370 informants (248 male and 122 female), most of them literate and ranging in ages from 24 to 104, were conducted with village heads, edible insect farmers, edible insect collectors, elderly people, educated youths, homemakers and traditional healers. The informants were asked about the whole insect or parts used for treating various ailments emphasizing the mode of preparation. The question on the strength of the family refers to the number of family members and data on income were only sought from insect vendors and may be used in a different publication. Folk stories, songs, proverbs and idioms containing references to insects abound, but will be the subjects of some publication in the future. Photographs and voucher specimens of species referred to in this paper were deposited at the Department of Zoology, Nagaland University, Lumami.

### Data analysis

Two quantitative tools (fidelity level and informant consensus factor) were used for data analysis. To evaluate the effectiveness and importance of a species for a particular disease, the fidelity level (FL) was used: FL (%) = Np/N × 100 (where, Np is the number of informants that claimed the use of an insect species to treat a particular disease and *N* is the number of informants that used the insects as a medicine for any given disease) [39]. To identify an insect species with high fidelity level, simple use mentions (UM), which refer to the mentions for one insect given by all the informants for a specific disease is cited [40].

To analyse the general use of insects, the informant consensus factor (ICF) was used. In order to use this tool, illness was classified into broad disease categories such as problems deemed (1) gastro-intestinal, (2) dermatological, (3) respiratory, (4) gynaecological/andrological, (5) pain, (6) fever (including malaria), (7) skeleton-muscular, (8) ophthalmological, (9) urological, (10) due to venomous animal bites, (11) cardiovascular, (12) to represent diabetes, (13) oncologic, (14) to have cultural filiations, and (15) to be due to other characteristics [16, 17]. The ICF was calculated according to [41] as the number of use citations in each category (Nur) minus the number of species used (Ns).

## Results

### Diversity of medicinal insects

The present study recorded 50 insect species belonging to 28 families and 11 orders for treating at least fifty different kinds of human ailments of which the most frequently cited ones amongst the ethnic groups were coughs, gastritis, rheumatoid arthritis, stomach ache and wound healing. Details regarding the medicinally used insect species are given in Table 1. The latter includes the insects’ local vernacular names, their habitats, the parts used as well as reasons for their uses. In addition to the disease category that the therapeutic insects are used for, tribal preferences/utilizations are also mentioned and any specific therapeutic knowledge is emphasized.

**Table 1:** Insect species used by the ethnic groups in Nagaland for treating various human health conditions

S.No	Scientific name	Family	Local name (TU)	Habitat	Parts used	Diseases (use mentions)	Therapeutic knowledge	FL% (category)
	Order: Odonata							
1	*Crocothemis servilia servilia* Drury1773	Libellulidae	Khapfü/ Sothinogo (AN, C) Note: adults possess different names	Aquatic	Nymph	Body ache (39); cold (30); vision (25); operational wound healing (12)	1. Boiled nymphs are eaten 2. Decoction of boiled nymphs are orally administered	60.9 (7); 46.8 (3); 39.1(8); 18.7 (2)
2	*Diplacodes trivialis* Rambur 1842							
3	*Neurothemis fulvia* Drury 1773							
4	*Orthetrum pruinosum neglectum* (Burmeister 1839)							
						Conjunctivitis (33)	Fumes of boiled nymphs are allowed to get in the eyes (twice daily)	51.5 (8)
5	*Orthetrum sabina sabina* Drury 1770							
6	*Orthetrum triangulare (*Selys 1878)							
7	*Pantala flavescens* Fabr. 1798							
8	*Potamarcha congener* Rambur 1842							
	Order: Orthoptera							
9	*Melanoplus* sp*.*	Acrididae	Phvütyara (L)	Paddy fields	Adult	Indigestion (25)	Boiled or roasted grasshoppers are eaten for quick recovery	51.0 (1)
						Diabetes (23)	Roasted grasshoppers are eaten to control blood sugar level	46.9 (12)
10	*Tarbinskiellus portentosus* (Lichtenstein 1796)	Gryllidae	Shungrü (L, C)	Burrowing	Adult	Headache (14); indigestion (27); loose motion (25); malaria (7)	Boiled or roasted crickets are eaten for quick recovery	21.5 (6); 41.5 (1); 38.4 (1); 10.8 (6)
11	*Gryllus* spp*.*	Gryllidae	Panto (L)	Wild	Adult	Dysentery (32)	Boiled or roasted crickets are eaten for quick recovery	50 (1)
12	*Pseudophyllus titan* White 1846	Tettigonidae	Satuqhu (S)	Wild	Adult	Fever (6)	Roasted katydids are orally administered to young children	31.6 (6)
13	*Elimaea securigera* Brunner von Wattenwyl 1878	Tettigonidae	Kozüvire (A, AN, C, K, L, S)	Paddy fields	Adult	Nutrient supplement (115)	Cooked katydids are eaten to strengthen the body	58.7 (15)
	Order: Mantodea							
14	*Tenodera sinensis* Saussure 1871	Mantidae	Shopile (L, S)	Wild	Adult	Warts (19)	An adult mantis is allowed to masticate on warts	52.8 (2)
15	*Hierodula coarctata* Saussure 1869	Mantidae	Hinube (AN)	Wild	Adult	Enuresis (8)	Roasted mantises are orally administered to young children	42.1 (9)
	Order: Blattodea							
16	*Epilampra* sp.?	Blaberidae	A	Aquatic	Nymph,adult	Appetizer (6), bloating (6)	Raw or roasted cockroaches are eaten	20.0 (15); 20.0 (1)
17	*Macrotermes* sp.	Termitidae	Holum (A, K, L, S)	Burrowing	Adult	Nutrient supplement (118)	Fried termites are eaten	62.4 (15)
	Order: Phasmatodea							
18	*Carausius* sp*.*	Lonchodidae	Hanga (S)	Wild	Adult	Calluses, warts (5)	Paste of stick insect is externally applied for quick removal	15.2 (2)
						Prickling spines (5)	Adult stick insect is rubbed for removal	15.2 (2)
	Order: Hemiptera							
19	*Darthula hardwickii* (Gray 1832)	Aetalionidae	Muzwü (C)	Wild	Nymph	Body ache (10); jaundice (6)	Boiled nymphs are eaten	31.3 (7); 18.8 (6)
20	*Lethocerus indicus* (Lepeletier & Serville 1825)	Belostomatidae	Khozhü/ Khutsia (AN, C)	Aquatic	Adult	Blood purifier (12); gastritis (13)	Decoction of boiled nymphs is orally administered daily for two to three days	11.9 (11); 12.9 (1)
						Dry coughs (48); diarrhoea (20); wound healing (13)	Soup of boiled bugs is orally administered for quick healing	47.5 (3); 19.8 (1); 26.7 (1)
						Rheumatoid arthritis (11)	Soup of boiled bugs is orally administered once daily	10.9 (7)
						Stomach ache (27)	1. Soup of boiled bugs is orally administered 2. Fumes of boiled bugs are inhaled for quick recovery	12.9 (2)
21	*Notobitus meleagris* Fabr. 1787	Coreidae	Azüpaza (S)	Wild	Adult	Stomach ache (25)	Roasted stink bugs are eaten for quick healing	46.3 (1)
22	*Aspongopus nepalensis* Westwood 1837	Dinidoridae	Tsseni (L)	Wild	Adult	Jaundice (11)	Boiled stink bugs are eaten twice daily for a week	34.4 (6)
23	*Coridius singhalanus* Dist. 1900	Dinidoridae	Longgea (C, L, S)	Wild	Adult	To induce lactation (6); jaundice (33); malaria (8)	Cooked stink bugs are eaten daily for a week	7.6 (4); 41.8 (6); 10.1 (6)
24	*Laccotrephes ruber* L. 1764	Nepidae	Segokikha (AN, C)	Aquatic	Adult	Blood purifier (12)	Decoction of boiled nymphs is orally administered daily for two to three days	21.4 (11)
						Gastritis (13)	Decoction of boiled nymphs is orally administered daily for a month	23.2 (1)
						Diarrhoea (18); stomach ache (9); wound healing (20)	Soup of boiled bugs is orally administered for quick healing	32.1 (1); 16.1 (1); 35.7 (2)
						Rheumatoid arthritis (11)	Soup of boiled bugs is orally administered once daily	19.6 (7)
25	*Udonga montana* Distant 1900	Pentatomidae	Shothapü (C)	Wild	Adult	Analgaesic (28); operational wound healing (20)	Boiled stink bugs are eaten for quick healing	87.5 (5); 35.7 (2)
						Coughs (22)	Roasted stink bugs are eaten for quick recovery	68.8 (3)
						Diabetes (10)	Roasted stink bugs are eaten to control blood sugar level	31.3 (12)
	Order: Neuroptera							
26	*Myrmeleon* spp*.*	Myrmeleontidae	Kudushürhu (AN, C)	Sandy	Larva	Boils (5); warts (24)	Paste of antlion is externally applied to cure boils and warts	11.9 (2); 57.1 (2)
	Order: Coleoptera							
27	*Cybister limbatus (*Fabr. 1775)	Dytiscidae	Dzübolo (AN)	Aquatic	Adult	Diarrhoea (30)	Boiled diving beetles are eaten for quick recovery	46.9 (1)
28	*Cybister tripunctatus lateralis (*Fabr. 1775)							
29	*Hydrophilus caschmirensis* Redtenbacher 1846	Hydrophilidae	Khujü (AN, C)	Aquatic	Adult	Operational wound healing (20)	Cooked water beetles are eaten for quick healing	20.0 (2)
30	*Mylabris* sp.	Meloidae	Chupnanutsü (A, AN, S)	Wild	Adult	Blisters (8); warts (33)	Blister beetle extract is externally applied	28.6 (2); 100 (2)
31	*Batocera rubus* L.1775	Cerambycidae	Loyie (ALL)	Trees	Larva	Analgaesic (79); diarrhoea (16)	Decoction of boiled larvae are orally administered	44.9 (5); 9.1 (1)
32	*Batocera parryi* Hope 1845					Aphrodisiac (10); malaria (12); joint pain (20); stomach ache (17); typhoid (8)	Soup of boiled larvae are orally administered	5.7 (4); 6.8 (6 ); 11.4 (7); 9.7 (1); 4.5 (6)
33	*Batocera rufomaculata* De Geer 1775							
34	*Orthosoma brunneum* (Forster 1771)	Cerambycidae	Lewo (ALL)	Trees	Larva	Analgaesic (79); diarrhoea (16)	Decoction of boiled larvae are orally administered	27.4 (5); 5.6 (1)
						Aphrodisiac (10); malaria (6); stomach ache (17); typhoid (8)	Soup of boiled larvae are orally administered	3.5 (4); 4.2 (6); 5.9 (1); 2.8 (6)
						Asthma (10); coughs (10)	Roasted larvae are orally administered	3.5 (3); 3.5 (3)
	Order: Hymenoptera							
35	*Apis cerana indica (*Fabr.1798)	Apidae	Tukhrü (ALL)	Wild	Honey	Blood pressure (14); cholera (14); cold (110); common illness (163); cough (131); diarrhoea (75); hangover (8); sinusitis (11); vertigo (213)	One table spoonful of honey is mixed in a cup of warm water and orally administered for quick recovery	3.8 (11); 3.8 (6); 29.7 (3); 44.1 (15); 35.4 (3); 25.3 (1); 2.2 (15); 3.0 (3); 57.6 (15)
					Honey	Mouth ulcers (57)	Raw honey is applied on affected parts	15.4 (2)
					Honey	Asthma (14); cancer (4); gastritis (23)	One table spoonful of honey is mixed in a cup of warm water and orally administered once daily	3.8 (3); 1.1 (13); 6.2 (1)
					Larva, pupa	Nutrient supplement (105)	Boiled/cooked larvae and pupae are eaten	28.4 (15)
					Honey/bee comb	Bone fractures (19); body ache (7); facial (12); wound healing (117)	1. Raw honey is externally applied 2. Poultice of bee comb is externally applied	5.1 (7); 1.9 (7); 3.2 (2); 31.6 (2)
					Honey	Conjunctivitis (22)	One or two drops of honey is applied on the eyes twice daily	5.9 (8)
					Adult	Headache (8); joint pain (7)	Bee sting on target points on the body	2.2 (6); 1.9 (5)
					Honey/bee comb	Pneumonia (18)	1. Honey is mixed with turmeric powder and externally applied on the chest (as per requirement) 2. Poultice of bee comb is externally applied on the chest	4.9 (3)
					Honey/bee comb/wax	Stomach ache (75)	1.One table spoonful of honey is mixed in a cup of warm water is orally administered 2. Poultice of bee comb/wax is orally administered	20.3 (1)
36	*Apis dorsata dorsata* (Fabr. 1793)	Apidae	Khwize (AN, C, L, S)	Wild	Honey/bee comb	Appetizer (12); cold (70); coughs (91); diarrhoea (78); vertigo (91)	1. One table spoonful of honey is mixed in a cup of warm water and orally administered 2. A small piece of bee comb is eaten or dissolved in a cup of warm water and orally administered	5.7 (15); 33.3 (3); 43.3 (3); 37.1 (1); 43.3 (15)
					Honey	Common illness (35)	One table spoonful of honey is mixed in a cup of warm water and orally administered for quick recovery	16.7 (15)
					Adult	Oedema (6)	1. Soup of boiled adult is orally administered 2. Paste of boiled adults are externally applied on affected body parts	2.9 (15)
					Larva, pupa	Heart disease (12); nutrient supplement (70)	Cooked larvae and pupae are orally administered	5.7 (11); 33.3 (15)
37	*Apis laboriosa* Smith 1871	Apidae	Liuniutso (C, L)	Wild	Honey/bee comb	Appetizer (12); cold (56); coughs (91); diarrhoea (38); vertigo (56)	1. One table spoonful of honey is mixed in a cup of warm water and orally administered 2. A small piece of bee comb is eaten or dissolved in a cup of warm water and orally administered	10.9 (15); 50.9 (3) 82.7 (3); 34.5 (1); 50.9 (15)
					larva, pupa	Nutrient supplement (61)	Cooked larvae and pupae are orally administered	55.5 (15)
38	*Apis florea* Fabr. 1787	Apidae	Rhontso (C, L)	Wild	Honey/bee comb	Appetizer (12); cold (56); coughs (91); diarrhoea (38); vertigo (56)	1. One table spoonful of honey is mixed in a cup of warm water and orally administered 2. A small piece of bee comb is eaten or dissolved in a cup of warm water and orally administered	10.9 (15); 50.9 (3); 82.7 (3); 34.5 (1); 50.9 (15)
					Larva, pupa	Nutrient supplement (61)	Cooked larvae and pupae are orally administered	55.5 (15)
39	*Lepidotrigona arcifera* Cockerell 1929	Apidae	Ruyo (ALL)	Wild	Honey	Analgaesic (32); appetizer (12); blood pressure (34); cancer (12); chest pain (18); coughs (75); gastritis (37); common illnesses (90); heart disease (12); vertigo (69)	One table spoonful of honey is mixed in a cup of warm water and orally administered	8.6 (5); 3.2 (15); 9.2 (11); 3.2 (13); 4.9 (3); 20.3 (3); 10(1); 24.3 (1); 3.2 (11); 18.6 (15)
40	*Lophotrigona canifrons* (Smith 1857)							
					Honey/nest entrance	Diarrhoea (109)	1. One table spoonful of honey is mixed in a cup of warm water and orally administered 2. A small piece of nest entrance is dissolved in a cup of warm waterand orally administered	29.5 (1)
					Honey/nest entrance	Bone fracture (42); body ache (11); dog bite (16); oedema (11); joint pain (17); mouth ulcers (34); pneumonia (12); skin burns (59); snake bite (72); wound healing (177); tooth aches (11)	1. Honey is externally applied on affected body parts 2. Paste or poultice of nest entrance is externally applied	11.4 (7); 3.0 (7); 4.6 (7); 4.3 (2); 3.0 (15); 9.2 (2); 3.2 (3);15.9 (2); 19.5 (10); 47.8 (2); 3.0 (15)
					Honey	Conjunctivitis (33)	One or two drops of honey is applied on the eyes twice daily	8.9 (8)
					Honey/nest entrance	Easy labour (61)	1. One table spoonful of honey is mixed in a cup of warm water and orally administered 2. Small piece of nest entrance is dissolved in a cup of warm water and orally administered 3. Paste or poultice of nest entrance is externally applied on the belly	16.5 (4)
					Nest entrance	Ritual (10)	A piece of nest entrance is carried along to ward off evil spirits	2.7 (14)
41	*Oecophylla smaragdina* Fabr. 1775	Formicidae	Tssonthen (A, L, K, S)	Trees	All	Analgaesic (8); coughs (13)	1.Cooked ants are orally administered 2. Decoction of boiled ants are orally administered for quick recovery	3.9 (5); 6.3 (3)
					Adult	Malaria (21); typhoid (17)	Decoction of boiled ants are orally administered daily for a week	10.2 (6); 8.3 (6)
					Adult	Oedema (9)	Adult ants are made to sting on swollen body parts	4.4 (15)
					Adult	Fever (19); headache (13)	Ants are cooked with local spices (garlic and ginger) and orally administered	9.3 (6); 6.3 (6)
					Adult	Sinusitis (67)	1. Fumes of ants are inhaled thrice daily 2. Boiled ants are orally administered 3. Soup of boiled ants are orally administered	32.7 (3)
42	*Provespa barthelemyi* (Byusson 1905)	Vespidae	Angüi (S)	Wild	Adult	Analgaesic (23)	Wasp sting on affected body parts	38.9 (7)
					All	Insomnia (5)	1. Boiled wasps are orally administered 2. Soup of boiled wasps are orally administered	8.5 (15)
43	*Vespa mandarinia* Smith 1852	Vespidae	Nati (ALL)	Burrowing	Larva, pupa	Diabetes (10)	1. Boiled wasps are orally administered 2. Soup of boiled ants are orally administered	2.7 (12)
					Larva, pupa	Nutrient supplement (334)	Raw/cooked larvae and pupae are eaten	90.2 (15)
					All	Sharpens memory (7)	Boiled/cooked wasps are eaten	1.9 (15)
44	*Vespa tropica tropica* (L. 1758)	Vespidae	Akizu (S)	Trees	Adult	Oedema (27)	Roasted adults are consumed or externally applied on affected body parts	38.6 (15)
					Larva, pupa	Nutrient supplement (49)	Raw/cooked larvae are eaten	70.0 (15)
	Order: Lepidoptera							
45	*Cossus* sp*.*	Cossidae	Loyie (ALL)	Trees	Larva	Analgaesic (62); bloating (10); nausea(11)	Boiled/cooked larvae are eaten for quick recovery	22.8 (5); 3.7 (1); 4.0 (1)
					Larva	Common illnesses (19)	Boiled/cooked larvae are orally administered	7.0 (15)
					Larva	Gastritis (19)	Boiled larvae are eaten daily	7.0 (1)
					Larva	Joint pain (40); rheumatoid arthritis (48)	1. Boiled/cooked larvae are eaten daily 2. Paste of raw larvae are externally applied on the affected body parts	14.7 (7); 17.6 (7)
					Larva	Asthma (11)	Decoction of boiled larvae is orally administered daily	4.0 (3)
					Larva	Diabetes (7)	Roasted larvae are eaten to maintain blood sugar level	2.6 (12)
					Larva	Cough (13); general weakness (11); stomach ache (21)	1. Decoction of boiled larvae is orally administered 2. Roasted larvae are eaten	4.8 (3); 4.0 (15); 7.7 (1)
					Larva	Typhoid (5)	Decoction of boiled larvae is orally administered for a week	1.8 (6)
					Larva	Blood purifier (24)	1. Boiled larvae are eaten 2. Soup of boiled larvae is orally administered	8.8 (11)
					Larva	Malaria (5)	1. Boiled larvae are eaten in alternate days for a week 2. Two to three piece of raw larvae are eaten in alternate days for a week	1.8 (6)
46	*Omphisa fuscidentalis* Hampson 1896	Crambidae	Keselu (AN, A)	Wild	Larva	Analgaesic (29)	1. Cooked larvae are eaten 2. Decoction of boiled larvae is orally administered	36.7 (7)
47	*Erionata torus* Evans 1941	Hesperiidae	Niakirülo (AN, L)	Home garden	Larva	Aphrodisiac (7); snake bite (11)	1. Cooked larvae are eaten 2. Decoction of boiled larvae is orally administered	6.1 (4); 9.6 (9)
48	*Malacosoma* sp*.*	Lasiocampidae	Meshangra (L)	Trees	Larva	Analgaesic (3); joint pain (3)	1. Cooked larvae are eaten 2. Decoction of boiled larvae is orally administered	12.0 (7); 12.0 (7)
49	*Samia cynthia ricini (*Boisduval 1854)	Saturniidae	Eriapok (ALL)	Wild/Home garden	Larva	Analgaesic (16); blood pressure (11); diabetes (29)	1. Cooked larvae are eaten 2. Decoction of boiled larvae is orally administered	4.7 (5); 32. (11); 8.6 (12)
					Larva	Nutrient supplement (164)	Cooked larvae are eaten	48.4 (15)
	Order: Diptera							
50	*Tipula* spp*.*	Tipulidae	Sonhe (AN, C, KH)	Aquatic	Larva	Analgaesic (51)	Decoction of boiled larvae is orally administered	38.9 (5)
					Larva	Eyesight (5); fatigue (11)	Cooked larvae are eaten	3.8 (8); 8.4 (15)
					Larva	Measles (6)	1. Cooked larvae are eaten 2. Decoction of boiled larvae is orally administered	4.6 (6)

*TU* Tribe utilization, *A*-*Ao* AN-Angami, *Al* all tribes, *C* Chakhesang, *KH* Khiamnuingan, *K* Konyak, *L* Lotha, *S* Sumi

**Fig. 2:**
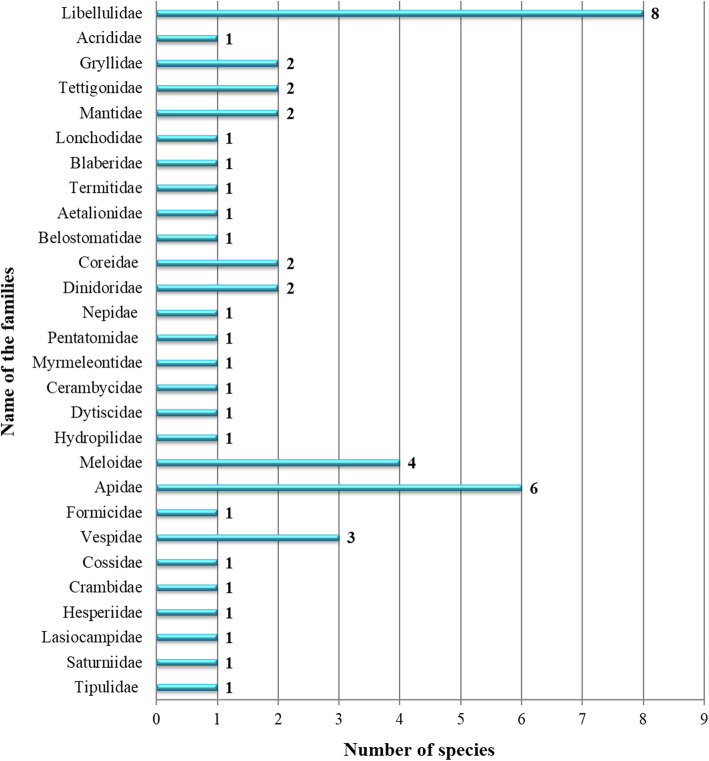
Graphical representation of the total number of families documented from the study

**Fig. 3:**
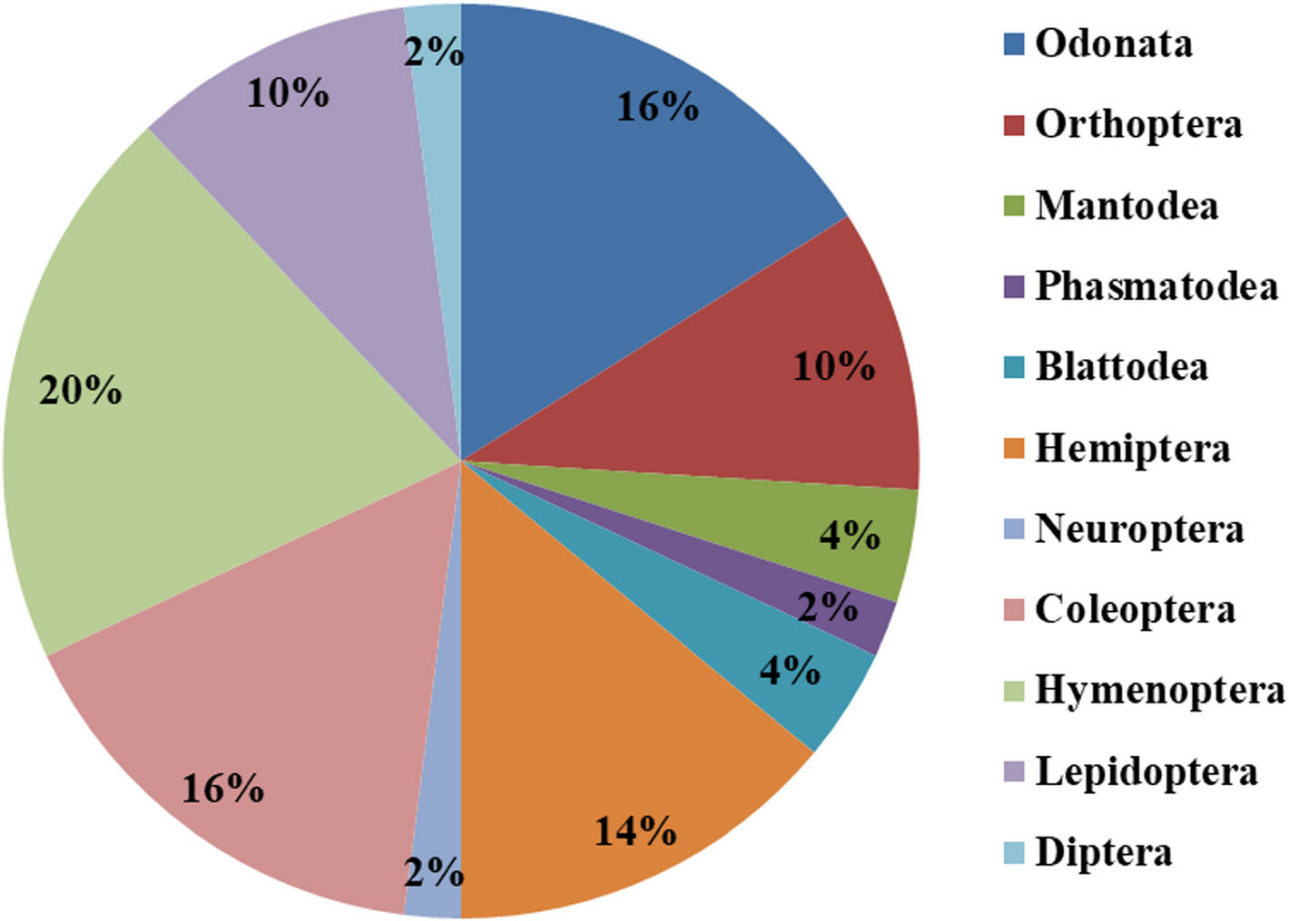
Diagrammatic representation showing the percentage contribution by each insect order

The dominant families reported in the study are the Libellulidae (16%), followed by Apidae (12%), Cerambycidae (8%) and Vespidae (6%) (Fig. 2). Orders represented in the study are Odonata (8 spp.; 16%), Orthoptera (5 spp.; 10%), Mantodea (2 spp.; 4%), Phasmatodea (1 sp.; 2%), Blattodea (2spp.; 4%), Hemiptera (7 spp.; 14%), Neuroptera (1 sp.; 2%), Coleoptera (8 spp.; 16%), Hymenoptera (10 spp.; 20%), Lepidoptera (5 spp.; 10%) and Diptera (1 sp.; 2%) (Fig. 3). It would, of course, have been desirable for the analysis to possess data on the total number of insect species known to the interviewees, but the region the survey was carried out is considered one of the remotest in India and according to the Zoological Survey of India a large number of insects of that part of India remains unrecognized and undescribed. Besides, the ‘species concept’ of the local people is very different from that used by scientific taxonomists.

**Fig. 4:**
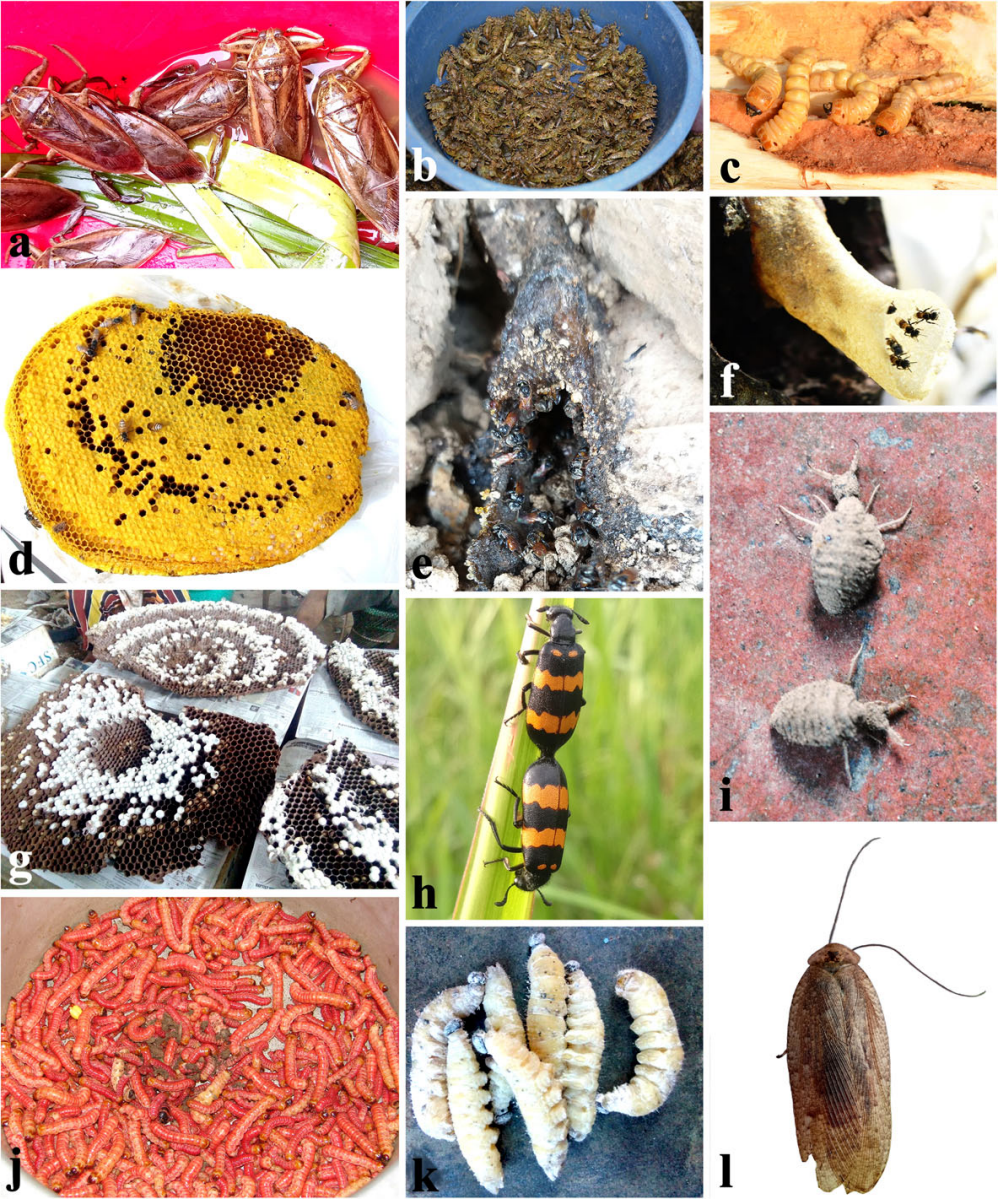
Certain medicinal insect and insect products of Nagaland. **a** Giant water bug *Lethocerus indicus*, **b** dragonfly nymphs, **c** large timber-boring larvae, **d** freshly harvested *Apis florea* bee comb, **e, f** nest entrances of stingless bees, **g** *Vespa mandarinia* comb sold at local market, Kohima district, **h** blister beetle *Mylabris* sp., **i** larvae of antlion *Myrmeleon* sp., **j** larvae of *Cossus* sp., **k** larvae of banana skipper *Erionata torus*, **l** *Epilambra* sp. cockroach

**Fig. 5:**
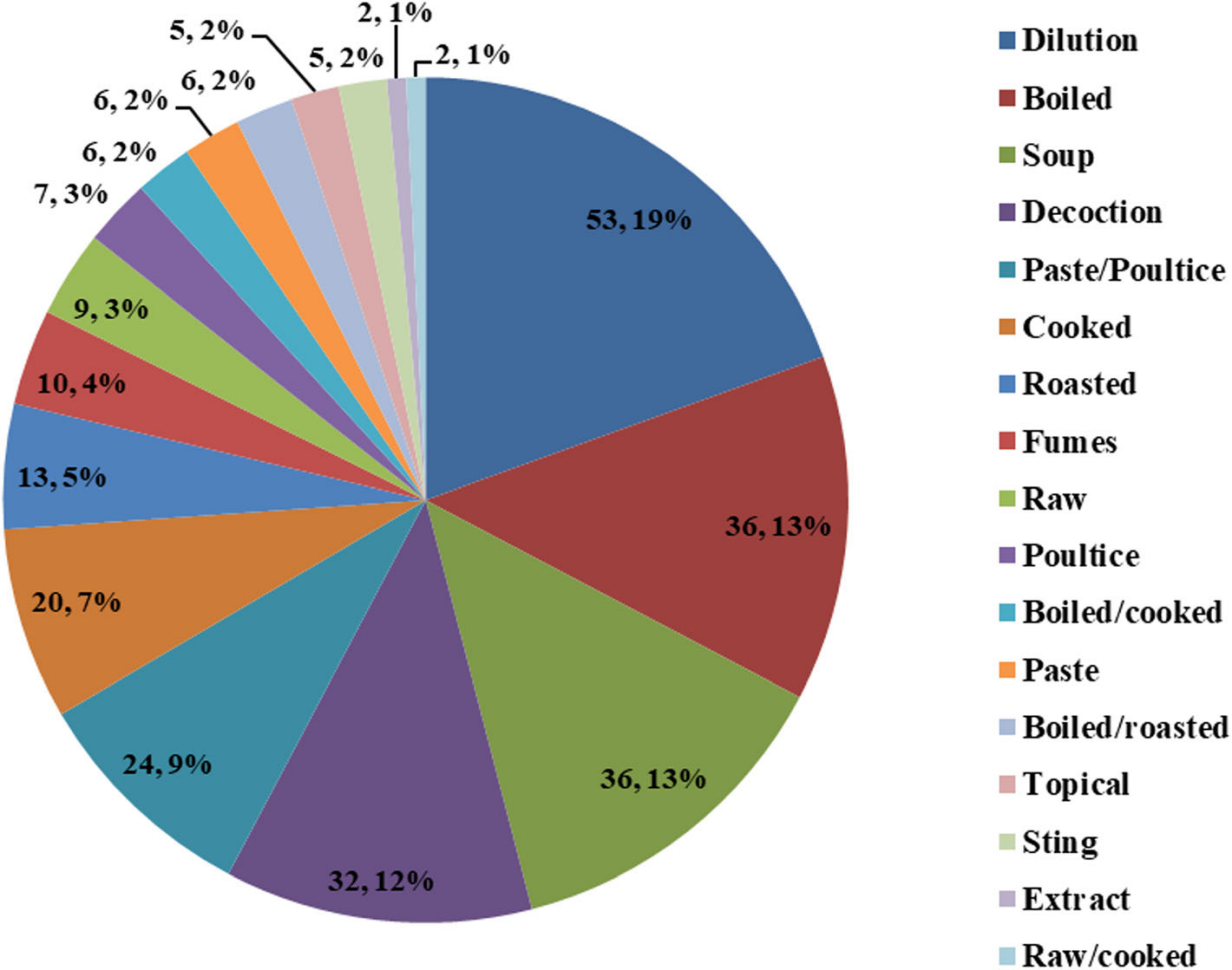
Percentage contribution of different preparation methods of medicinal insects

Important medicinal insect species are used in their larval, nymphal, pupal and adult stages or as by-products. Freshly harvested insects are preferred in traditional medicines and 100% of the informants have utilized at least one medicinal insect or its derived products in their life. Certain important medicinal insects reported are presented in Fig. 4. Of the 50 medicinal insects, 47 species were also highly appreciated as food [32] while 3 insect species (*Carausius* sp., *Myrmeleon* sp*.* and *Mylabris* sp.) were considered inedible and only meant to be used for topical application and to treat certain ailments like blisters, calluses and warts. Medicinal insects for treating human ailments are mostly used as a dilution (*n* = 53; 20%), boiled (*n* = 36; 13%), in a soup (*n* = 36; 13%), as a decoction (*n* = 32; 12%), as paste/poultice (*n* = 24; 9%) or in cooked form (*n* = 20; 7%). The percentage-wise contribution of the different preparation methods is presented in Fig. 5.

**Fig. 6:**
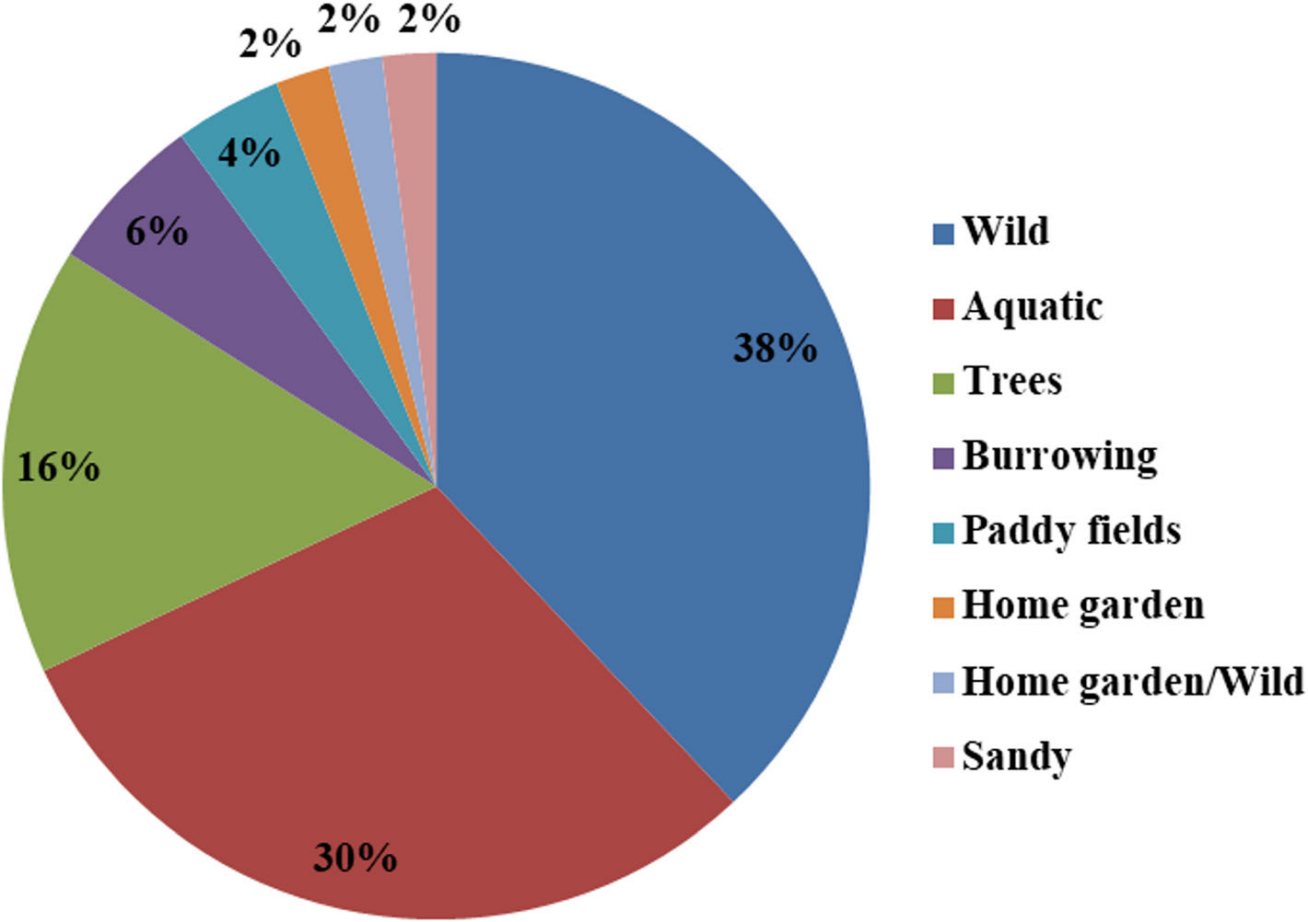
Habitats of medicinal insects reported in Nagaland

Medicinal insect species that are reported in the present study are mostly collected from the wild terrestrial (*n* = 19; 38%) and wild aquatic environments (*n* = 15; 30%). Trees (*n* = 8; 16%), underground burrows (*n* = 3; 6%), paddy fields (*n* = 2; 4%) and one species each obtained from home garden, both home garden and wild, and sandy habitats were also mentioned (Fig. 6). The insects with major numbers of use-indications for any disease were *Vespa mandarinia* (334), *Apis cerana indica* (213), *Lepidotrigona arcifera* (177), *Lophotrigona canifrons* (177), *Samia cynthia ricini* (164), *Macrotermes* sp. (118), *Elimaea securigera* (115), *Apis dorsata dorsata* (91), *Apis laboriosa* (91) and *Apis florea* (91). Insect species with the most citation-uses in Naga folk medicine were the common Indian honey bee *Apis cerana indica* and the stingless bees *Lepidotrigona arcifera* and *Lophotrigona canifrons*.

### Fidelity level

In terms of fidelity level value (Table 1), *Melanoplus* sp. (FL = 51%) turns out the most important species for the gastrointestinal category particularly preferred by the Lothatribe. The value indicates that indigestion is the most common ailment treated. *Mylabris* sp. (FL = 100%) is the most important species for the dermatological category with warts as an ailment receiving the majority of treatment amongst the Ao tribe besides Angami and Sumi tribes. All seven ethnic groups consider *Apis laboriosa* and *Apis florea* (FL = 82.7%) to be the most important species in treating the respiratory problems with coughs being the most common ailment to be treated, while the stingless bees *Lepidotrigona arcifera* and *Lophotrigona canifrons* (FL = 16.5%) are regarded as the most important species among the Angami and Chakhesang tribes for gynaecological/andrological problems with easy labour as a significant use category. *Udonga montana* (FL = 87.5%) and *Coridius singhalanus* (FL = 41.8%) were most important in connection with pain and fever, respectively, with analgesic and jaundice being the dominant ailments to be treated. Dragonfly nymphs (FL = 60.9%) were the leading insects in the skeleton-muscular problem category characterized by body aches as well as for ophthalmological problems like conjunctivitis (FL = 51.5%).

The praying mantis *Hierodula coarctata* (FL = 42.1%) was the choice species in the urological category with enuresis as the significant ailment whereas, the stingless bees *Lepidotrigona arcifera* and *Lophotrigona canifrons* (FL = 19.5%) were the most important species in connection with venomous animal bites, in which snake bites featured as the main and most serious problem. The giant water scorpion *Laccotrephes ruber* (FL = 21.4%) was the most important species in the cardiovascular category given its assumed blood purifier properties, while in the diabetes category *Melanoplus* sp. (FL = 46.9%) came out as an important species utilized, only, however, by the Lotha tribe. In the oncologic and cultural filiation’s category, the stingless bees *Lepidotrigona arcifera* and *Lophotrigona canifrons* (FL = 3.2%; FL = 2.7%) once again reached the number one position of the important species and it was emphasized by the informants that the honey of stingless bees kept for 7 years or more (possibly undergoing some fermentation) was particularly medicinal.

### Informant consensus factor

It is evident from the informant consensus factor (ICF) that there are some parallel usages of medicinal insects among the seven ethnic groups. The parallel use of insect species may be due to coincidence, similar criteria for selecting insects or shared information on the potential usefulness of a species [41]. The similarities and differences with regard to the utilization of certain kinds of medicinal insects reported in the present study suggests that cultures differing in traditions and languages interact with each other, but also develop their own preferences. Of the fifty medicinal insects, the maximum number of species is used for gastro-intestinal, respiratory and dermatological problems (Table 2). In comparison with Angami, Ao, Chakhesang, Khiamnuingan, Konyak and Sumi tribes, the Lotha tribe showed the highest ICF values. A detailed summary of the ICFs for the seven ethnic groups is presented in Table 3. The ICF values for the Angami tribe indicate that fever, diabetes, oncologic and the disorders of the urological category scored the highest (ICF = 1.00) while the ophthalmological category (ICF = 0.84) scored the lowest consensus value when compared with other sickness categories.

**Table 2:** Important insect species for gastrointestinal and respiratory problems

Gastrointestinal (FL %)	Respiratory (FL %)	Dermatological (FL %)
*Melanoplus* sp*.* (51.0)	*Apis laboriosa* (82.7)	*Mylabris* sp. (100.0)
*Gryllus* spp*.* (50.1)	*Apis florea*(82.7)	*Myrmeleon* spp*.* (57.1)
*Cybister limbatus* (46.9)	*Udonga montana* (68.8)	*Lepidotrigona arcifera* (47.8)
*Cybister tripunctatus lateralis* (46.9)	*Lethocerus indicus* (47.5)	*Lophotrigona canifrons* (47.8)
*Notobitus meleagris* (46.3)	*Crocothemis servilia servilia* (46.8)	*Laccotrephes ruber* **(**35.7)
*Tarbinskiellus portentosus* (41.5)	*Diplacodes trivialis* (46.8)	*Udonga montana* (35.7)
*Apis dorsata dorsata* (37.1)	*Neurothemis fulvia* (46.8)	*Apis cerana indica* (31.6)
*Apis laboriosa* (34.5)	*Orthetrum pruinosum neglectum* (46.8)	*Lethocerus indicus* (26.7)
*Apis florea* (34.5)	*Orthetrum sabina sabina* (46.8)	*Hydrophilus caschmirensis* (20.1)
*Laccotrephes ruber* (32.1)	*Orthetrum triangulare* (46.8)	*Crocothemis servilia servilia* (18.7)
*Lepidotrigona arcifera* (29.5)	*Pantala flavescens *(46.8)	*Diplacodes trivialis*(18.7)
*Lophotrigona canifrons* (29.5)	*Potamarcha congener* (46.8)	*Neurothemis fulvia* (18.7)
*Apis cerana indica* (20.3)	*Apis dorsata dorsata* (43.3)	*Orthetrum pruinosum neglectum* (18.7)
*Epilampra* sp. (20)	*Apis cerana indica* (35.4)	*Orthetrum sabina sabina* (18.7)
*Lethocerus indicus* (19.8)	*Oecophylla smaragdina* (32.7)	*Orthetrum triangulare triangulare* (18.7)
*Batocera rubus* (9.7)	*Lepidotrigona arcifera* (20.3)	*Pantala flavescents* (18.7)
*Batocera parryi* (9.7)	*Lophotrigon acanifrons* (20.3)	*Potamarcha congener* (18.7)
*Batocera rufomaculata* (9.7)	*Cossus* sp*.* (4.8)	*Carausius* sp*.* (15.2)
*Cossus* sp*.* (7.7)	*Orthosoma brunneum* (3.5)	
*Orthosoma brunneum* (5.9)

**Table 3:** Informant consensus factor of every human health conditions

Category of indigenous uses	No. of species (Ns)							No. of use reports (Nur)							ICF						
	AN	A	C	KH	K	L	S	AN	A	C	KH	K	L	S	AN	A	C	KH	K	L	S
Gastrointestinal problems	10	3	8	5	7	8	6	162	22	60	28	52	141	142	0.94	0.90	0.88	0.85	0.88	0.95	0.96
Dermatological problems	7	4	14	3	3	4	6	125	64	83	43	33	80	128	0.95	0.95	0.84	0.95	0.93	0.96	0.96
Respiratory problems	10	2	10	–	3	5	4	64	24	126	-	80	162	65	0.85	0.95	0.92	–	0.97	0.97	0.95
Gynaecologic/andrologic	2	–	3	–	–	1	4	40	–	27	–	–	7	10	0.97	–	0.92	–	–	1.00	0.66
Pain	7	5	2	–	1	6	8	85	16	51	–	8	40	131	0.92	0.73	0.98	–	1.00	0.87	0.94
Fever (including malaria)	1	–	8	–	–	3	2	8	–	51	-	–	43	27	1.00	–	0.86	–	–	0.95	0.96
Skeleto-muscular problems	10	–	6	2	–	8	2	79	–	65	11	–	74	42	0.88	–	0.92	0.90	–	0.90	0.97
Ophthalmological	10	–	–	–	–	2	–	60	–	-	–	–	33	-	0.84	–	-	–	–	0.96	–
Urological	1	–	–	–	–	-	–	8	–	-	–	–	-	-	1.00	–	-	–	–	–	-
Poisonous animal bites	3	2	1	–	–	-	–	48	17	18	–	–	-	-	0.95	0.93	1.00	–	–	–	-
Cardiovascular	4	–	3	–	–	3	3	35	–	35	–	–	48	31	0.91	–	0.94	–	–	0.95	0.93
Diabetes	1	–	1	–	–	2	2	11	–	10	–	–	41	17	1.00	–	1.00	–	–	0.97	0.93
Oncologic	1	–	–	–	–	2	–	4	–	-	–	–	12	-	1.00	–	–	–	–	0.90	–
Cultural filiations	–	2	–	–	–	–	–	–	10	-	–	–	-	-	-	0.88	–	–	–	–	–
Others	8	8	10	5	6	10	10	181	172	141	102	152	321	380	0.96	0.95	0.93	0.96	0.96	0.97	0.97

It is also evident that dermatological and respiratory problems (ICF = 0.95) had the highest ICF values among the Ao tribe while the pain category (ICF = 0.73) received a lower consensus. Categories like diabetes and venomous animal bites recorded the highest value (ICF = 1.00) amongst the Chakhesang tribe, while the dermatological category (ICF = 0.84) showed a lower consensus compared with the other sickness categories. However, for the Khiamnuingan tribe, the dermatological category recorded the highest value (ICF = 0.95). The pain (ICF = 1.00) and gynaecological categories (ICF = 1.00) recorded the highest values among the Konyak and Lotha tribes, respectively, whereas skeleto-muscular problems, with an ICF of 0.97, yielded the highest value amongst the Sumi tribe.

### Diversity of medicinal insects among the ethnic groups

**Fig. 7:**
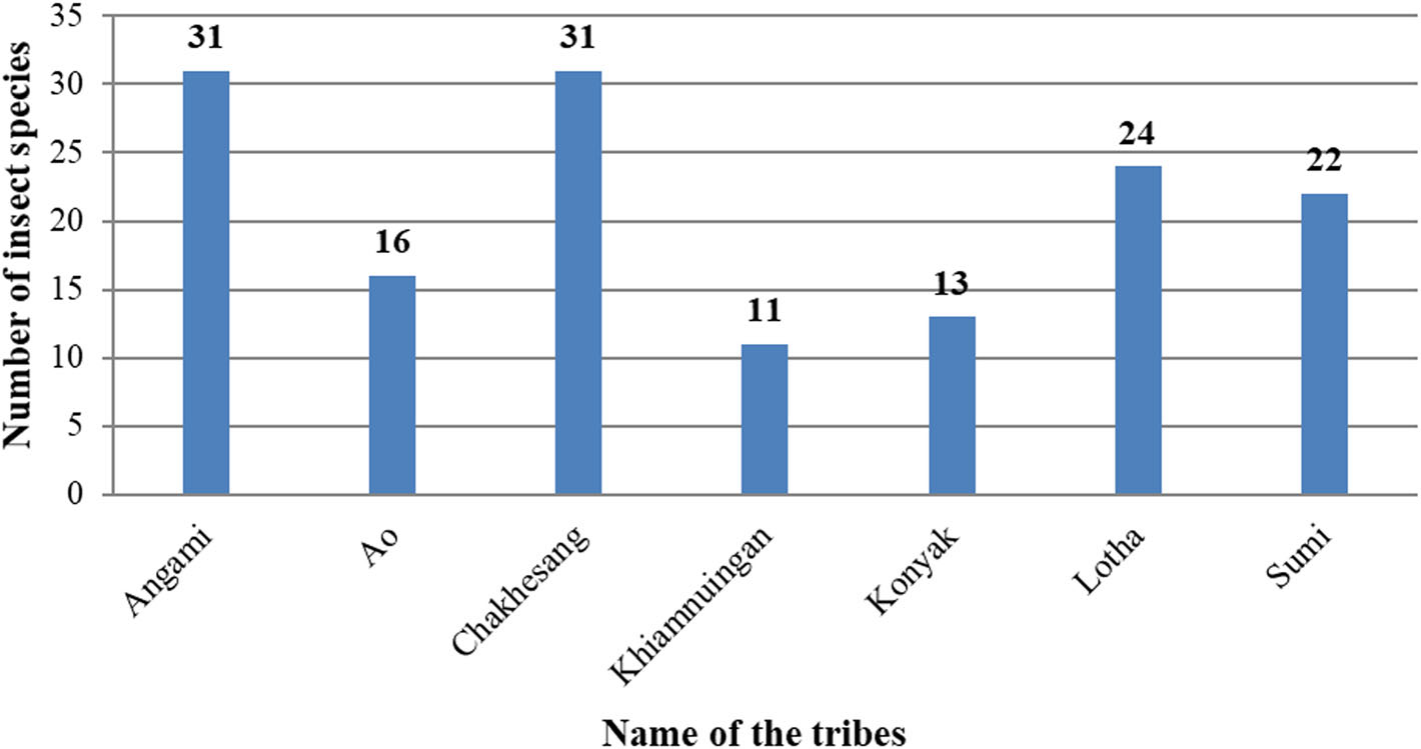
Diversity of medicinal insects utilized by each tribe

**Fig. 8:**
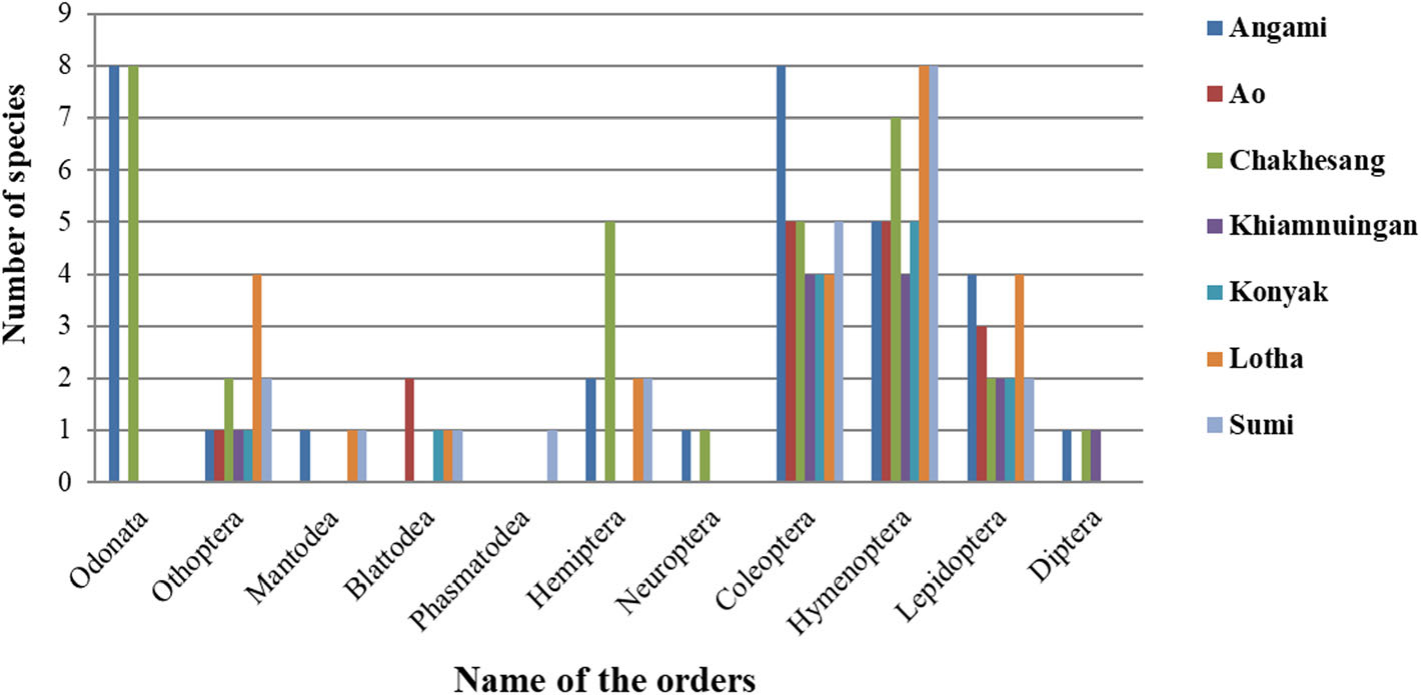
Order-wise distribution of insect species among the seven tribes

The present study reported a total of 50 medicinal insects. However, not all of the insect species were utilized by all seven ethnic groups. Of the seven ethnic groups, the Chakhesang and Angami tribes use the maximum number of insects for therapy with 31 species followed by members of the Lotha tribe with 24 species; the least number of insect species used therapeutically is 11 by the Khiamnuingan tribe (Fig. 7).The order-wise distribution of medicinal insects among the seven ethnic groups is presented in Fig. 8. A given insect species may be used for different purposes by different ethnic groups. For instance, dragonfly nymphs are reported to be used by only two tribes (Angami and Chakhesang). But while the Angami tribals use dragonfly nymphs for treating body aches, cold and ophthalmological problems, Chakhesang use dragonfly nymphs for healing wounds.

The field cricket *Tarbinskiellus portentosus*, utilized by the Chakhesang and Lotha tribes, serve different medicinal purposes for these two tribes. While the Chakhesang use the cricket to treat malaria, the Lotha tribe uses it in connection with headaches and gastro-intestinal problems. Lotha and Sumi tribes have identical medicinal uses for the mantis *Tenodera sinensis*, namely for treating warts. Similarities among the Angami and Chakhesang tribes with regard to *Lethocerus indicus* have been reported whereby the bugs are used to treat gastro-intestinal problems. Besides gastro-intestinal problems, the Chakhesang tribe also use giant water bugs as a remedy for rheumatoid arthritis and wound healing. The dinidorid bug *Coridius singhalanus* is used by the Chakhesang, Lotha and Sumi tribes. However, while the Lotha and Sumi share the same traditional therapeutic knowledge (treating jaundice), the Chakhesang tribe uses the bugs for treating malaria and to increase milk production in lactating mothers. Angami and Chakhesang tribes share the therapeutic knowledge of *Laccotrephes ruber* being an important medicinal agent to treat gastro-intestinal problems. However, in addition, water scorpions are also used as a remedy for treating rheumatoid arthritis by the Chakhesang tribe.

All of the seven tribes share the therapeutic knowledge that involves the larvae of wood borers (*Batocera rubus*, *Batocera parryi*, *Batocera rufomaculata* and *Orthosoma brunneum*) as an analgesic and a remedy to treat gastro-intestinal problems. However, the Chakhesang tribe also uses them for treating malaria and typhoid whereas the Sumi tribe take them as an aphrodisiac. The larvae of the banana skipper *Erionata torus* are used as an aphrodisiac by the Lotha tribe, but the Angami tribe use them to reduce the effects of venomous animal bites. While the Angami and Chakhesang tribes share similar therapeutic knowledge with regard to crane flies of the genus *Tipula *as an analgesic, the Khiamnuingan tribals use the larvae for treating measles in children.

## Discussion

### Healing with insects: traditions of the Nagas and other Indian tribals

The list of medicinal insect species in the present study highlights the diversified insect use as traditional folk medicine among the tribal communities of Nagaland. There are similarities with other ethnic tribes in the world, generally, and different regions of the country, in particular, as bees (Hymenoptera) and their products, but furthermore beetles (Coleoptera) and bugs (Hemiptera), dominate the list of the therapeutic species. The relatively high importance of dragonflies (especially as nymphs), but also aquatic beetles, an aquatic cockroach and species of the Neuroptera, however, makes the Naga therapeutic use of insects stand out somewhat and may be related to the abundance of streams and rivulets in the state.

Insect and insect-derived products provide ingredients that have been a staple in traditional medicine for centuries in many parts of the world and although many of these ingredients still have not been evaluated experimentally, an increasing number of them have been identified and shown to have beneficial properties [8, 18, 19, 42]. Because of its antimicrobial, anti-bacterial, anticancer, anti-diabetic, anti-hypercholesterolemia, anti-inflammatory, antioxidant and wound healing properties [6, 42–49], Nagas consider honey to be an extremely important medicinal agent for treating a multitude of human ailments such as cancer, cholera, gastrointestinal problems, respiratory problems, ophthalmological problems, etc. Six different types of honey are used by the Naga tribes in wound healing and for treatment of other disorders such as infections and irritable bowel syndrome which is also reported elsewhere.

The present findings of an ophthalmological use and topical application of honey over deep wounds as well as the use of bee pupae and bee hive material to treat back pain, throat pain and menstrual disorders is in accordance with the tribal communities of Rajasthan in India and people elsewhere in the world [49–52]. The oral administration of honey and bee comb/wax of the species *Apis cerana indica*, *Apis dorsata* and *Apis florea*, practiced by nearly all Naga tribes in treating asthma, cancer, coughs, colds, diarrhoea, gastritis, mouth ulcer, skin diseases, stomach pains, symptoms of nausea and various respiratory diseases as well as labour pains, shows similarities to that of indigenous people from other parts of India [16, 18, 52–60] and indeed the world [5, 6].

The therapeutic practice to use adult ants (*Oecophylla smaragdina*) among various tribes in Nagaland for the treatment of coughs, fever, malaria, typhoid, oedema, sinus infections and as an analgesic has also been reported from Assam, Arunachal Pradesh, Tamil Nadu and Kerala [54–62]. These common uses are almost certainly due to the observation that pharmacologically active compounds with antioxidant, anti-arthritic and antimicrobial activities in the abdominal glands of the species [63] provide relieve of debilitating symptoms. There would, of course, also have been cases in which members of different tribes exchanged their therapeutic knowledge. An identical use of boiled dragonfly nymphs for wound healing has also, for example, been reported from the Meitei community of Manipur, a state of North-East India with a significant proportion of Naga inhabitants [18].

### Comparisons with other tribes and countries

The use of *Melanoplus* sp. to treat certain intestinal disorders and stink bugs as an analgesic and for remedying stomach aches and rheumatoid arthritis shows similarities with ethnic Mexican communities [46] and, therefore, represents a convergent and independently discovered therapeutic use of an insect. The oral administration of the timber borer (*Orthosoma* sp.) as an aphrodisiac by Nagas bears similarities to the practice of rural people in Mexico [64] but must have been discovered independently. The reported use of *Carausius* sp. to treat prickling spines and skin-related diseases as well as the topical application of *Myrmeleon* spp. to treat warts are shared with the traditional therapeutic practices of the ethnic communities of the North-East Indian state of Mizoram, which suggests contacts between Nagaland and Mizoram inhabitants [65]. The topical application of *Mylabris* sp. for treating blisters and warts reported in our study also features in the traditional Chinese and Korean medical pharmacopeia [12–14] and is almost certainly based on the widely known presence and function of cantharidin derived from the bodies of blister beetles [20, 66].

However, certain differences between the therapeutic uses of insects in Nagaland with those of other countries cannot be ignored. For instance, stick insects are used for treating calluses, warts and prickling spines by the Naga tribes, but in North Korea they are considered to contain potent healing powers and used to cleanse the body as well as to remove stomach upsets [14].While, *Gryllus* spp., *Aspongopus nepalensis* and *Oecophylla smaragdina* are used for treating dysentery, jaundice and as an analgesic to treat coughs, malaria, typhoid, oedema, fevers and headaches by the Nagas, their uses in the treatment of pneumonia, malaria and digestive problems, respectively, have been reported from the North-East Indian states of Tripura [58], Mizoram and Arunachal Pradesh [65].

The blister beetle *Mylabris* sp. is used by Nagas to treat blisters and warts, but the same species has been used to treat tumours or cancers in China [8]. Silkworms are used as an analgesic, nutrient supplement and for blood sugar control by the Nagas, while in Japan, they are used to cure a sore throat and nephritis [67]. Furthermore, *Hierodula coarcta*, *Tarbinskiellus portentosus*, *Gryllus* spp., *Cybister* sp., *Mylabris* sp., *Batocera* spp. and *Apis cerana indica* are used to treat dermatological problems, headaches, malaria and gastrointestinal problems by the various Naga tribes, but in China the aforementioned insect species are used to treat impotence, relieve body swellings, fever, foster detoxification, improve blood circulation, assist in managing rheumatism, menstrual symptoms and arthritic pains [68].

### For each malady one species or one species for all ills?

Based on these inconsistent findings, the questions one can ask are: how is it possible that one and the same species can be good for a multitude of illnesses and how can it be that there are treatments for identical disorders involving a variety of often taxonomically not even closely related species? Meyer-Rochow [5] has tried to answer these questions by pointing out that in the small bodies of insects a great variety of distinct compounds like metabolites, enzymes, hormones, neurotransmitters, etc. exist and that the different preparation and administration methods used by traditional healers could lead to an activation of different molecules in the therapeutic species, affecting different organs and exerting specific effects in the treated person. Since the chemical composition of insects stems either directly or in case of metabolites indirectly from the food that they have ingested during their growth phases, there is also the possibility that identical species, but occurring in different habitats and regions with differing soil and microclimatic conditions, obtained non-identical ingredients, which could then result in non-identical effects with regard to the potency of these insects’ various bioactive compounds.

The second question, namely that taxonomically unrelated species can be used to treat disorders or diseases in humans is likely to be related to the fact that insects can suffer from pathogenic agents like viruses, bacteria, fungi, etc. that also occur in vertebrates [69] and that in the cases of cancers, which invertebrates can also suffer from, proliferating cell lines, as in human cancers, are inevitably involved [70]. Insects have had hundreds of millions of years to evolve efficient defences against these common pathogens and it would have been ‘far more surprising to find that each group or even each species had evolved its own unique defence system fighting disease’ [5]. Thus, the explanations of how the therapies with dissimilar insect species can lead to identical outcomes and why on the other hand sometimes one and the same species can be used in connection with different disorders can be summarized in the following way: the treatment results very likely depend firstly on the food and habitat characteristics that the therapeutic species used in the treatment had experienced earlier in their growth phases; secondly, on the pre-treatment that the remedy had undergone before administration; and thirdly, on the details of how the remedy is to be administered to the suffering person. Thus, to record and identify not only the various therapeutic species but also from which region and habitat they came from as well as the particular ways in which they are meant to be used therapeutically is important. Sadly, this information is frequently missing and due to the secrecy that traditional healers often attach to their methods, the latter are ever so often not exactly easy to come by or even appreciated by those who manage to obtain them. Folk traditional knowledge, also referred to as ‘common sense’ [71], and its contribution through entomotherapy should therefore not prematurely be regarded as useless and outdated but has to be scientifically scrutinized. There is real potential that such studies can lead to the development of novel drugs and alternative treatment methods.

## Conclusion

Besides their use as a food item among the various ethnic groups in Nagaland, insects are also widely used therapeutically. Our documentation of at least 50 medicinal insects from seven tribes in Nagaland suggests that folk traditional knowledge is still a part of the tribal lives in the state. The list of medicinal insect species, many of which are reported for the first time in the present study, is evidence of a considerable diversity of therapeutically exploited insect species of the region and demonstrates that detailed analyses of certain bioactive substances of these species, deemed effective in treating illnesses and other disorders and given high fidelity levels by local users, could open up new prospects in the field of pharmacology.

## Supplementary Information

**Additional file 1: Supplementary file 1.** Demographic patterns of informants in the study area. **Supplementary file 2.** QUESTIONNAIRE FORMAT.

## Data Availability

All data generated or analysed during this study are included in this published article (and its supplementary information files).
