# Supplementary material for: Entomotherapy: a study of medicinal insects of seven ethnic groups in Nagaland, North-East India
Source: J Ethnobiol Ethnomed. 2021 Mar 22;17:17. doi: 10.1186/s13002-021-00444-1 (PMC7986042; doi:10.1186/s13002-021-00444-1)
Supplement: Supplementary file 1 — Additional file 1: Supplementary file 1. Demographic patterns of informants in the study area. Supplementary file 2. QUESTIONNAIRE FORMAT. [file 13002_2021_444_MOESM1_ESM.zip › Supplementary file 1.docx]

**Supplementary file 1.** Demographic patterns of informants in the study area.

| **Gender** | **Number of Informants** |
| --- | --- |
| Male | 248 (67%) |
| Female | 122 (33%) |
| **Age Group** |  |
| 25–34 | 60 (16%) |
| 25–44 | 58 (16%) |
| 45–54 | 59 (16%) |
| 55–64 | 55 (15%) |
| 65–74 | 57 (15%) |
| 75–84 | 58 (16%) |
| 85–94 | 18 (5%) |
| 95–104 | 5 (1%) |
| **Educational status** |  |
| Below high school | 230 (62%) |
| Above high school | 140 (38%) |
| **Informant status** |  |
| Key informant | 198 (54%) |
| General informant | 172 (46%) |
