# Supplementary material for: Entomotherapy: a study of medicinal insects of seven ethnic groups in Nagaland, North-East India
Source: J Ethnobiol Ethnomed. 2021 Mar 22;17:17. doi: 10.1186/s13002-021-00444-1 (PMC7986042; doi:10.1186/s13002-021-00444-1)
Supplement: Supplementary file 1 — Additional file 1: Supplementary file 1. Demographic patterns of informants in the study area. Supplementary file 2. QUESTIONNAIRE FORMAT. [file 13002_2021_444_MOESM1_ESM.zip › Supplementary file 1.pdf]

Supplementary file 1. Demographic patterns of informants in the study area.

**Gender**	**Number of Informants**
Male<br>Female	248 (67%)<br>122 (33%)
**Age Group**	
25–34<br>25–44<br>45–54<br>55–64<br>65–74<br>75–84<br>85–94<br>95–104	60 (16%)<br>58 (16%)<br>59 (16%)<br>55 (15%)<br>57 (15%)<br>58 (16%)<br>18 (5%)<br>5 (1%)
**Educational status**	
Below high school<br>Above high school	230 (62%)<br>140 (38%)
**Informant status**	
Key informant<br>General informant	198 (54%)<br>172 (46%)
