# Supplementary material for: Entomotherapy: a study of medicinal insects of seven ethnic groups in Nagaland, North-East India
Source: J Ethnobiol Ethnomed. 2021 Mar 22;17:17. doi: 10.1186/s13002-021-00444-1 (PMC7986042; doi:10.1186/s13002-021-00444-1)
Supplement: Supplementary file 1 — Additional file 1: Supplementary file 1. Demographic patterns of informants in the study area. Supplementary file 2. QUESTIONNAIRE FORMAT. [file 13002_2021_444_MOESM1_ESM.zip › SUPPLEMENTARY MATERIAL S1.docx]

**Supplementary file 2: QUESTIONNAIRE FORMAT**

Name of the informant: _____________________________________________
Age:

**25-34 35-44 45-54**

**55-64 65-74 75-84**

**85-94 95+**

Sex:

**Male**

**Female**

Community: ________________ Local dialect: _________________________

Qualification:

**Below high school**

**Above high school**

Occupation:

**Government servant**

**Farmer**

**Unemployed**

Informant:

**Key informant**

**General informant**

Contact No.: _________________ Strength of family: _____________________

Place of interview:
a. Name of place/ village: ______________________________________________
b. Location/ District: __________________________________________________
c. Other details: ______________________________________________________

d. Signature: _________________________________________________________

1. Do you eat insects?

**Yes**

**No**

2. If yes, can you name the edible insects consumed by you?

| **Sl.**  **no** | **Common**  **name** | **Local**  **name** | **Seasonal**  **availability** | **Edible**  **stage** | **Mode of**  **consumption** | **Therapeutic**  **value** | **Myths/**  **Folklore** |
| --- | --- | --- | --- | --- | --- | --- | --- |
| 1 |  |  |  |  |  |  |  |
| 2 |  |  |  |  |  |  |  |
| 3 |  |  |  |  |  |  |  |
| 4 |  |  |  |  |  |  |  |
| 5 |  |  |  |  |  |  |  |

3. If no, can you name the edible insects consumed by others?

a. Local name of the insects: ____________________________________

b. Name of the tribe consuming the insects: ________________________

c. Edible parts: _______________________________________________

d. Edible stage: ______________________________________________

e. Habitat of the insects: **Terrestrial/Aquatic/Burrowing**

f. Time of collection: _________________________________________

g. Seasonal availability: _______________________________________

h. Mode of collection: _________________________________________

i. Mode of preparation and consumption: __________________________

4. Are there any indigenous methods for capturing insects?

**Yes**

**No**

5. If yes, can you name them and explain in details?

6. Are chemicals used for capturing insects?

**Yes**

**No**

7. If yes,

a. Name of the chemical: _________________________

b. Name of the insect captured: ____________________

8. Do you preserve the edible insects for future consumption?

**Yes**

**No**

9. If yes, then how are they preserved?

**Fermented**

**Smoked**

**Sundried**

**As Pickle**

Others: **________________________________________________**

10. Is entomophagy (insect consumption) popular in the region?

**Yes**

**No**

11. If yes, what are the most popular edible insects? Name them.

12. How often do you take/consume insects?

**Everyday**

**Once in a week**

**Several days in a week**

**Never**

13. Are insects reared?

**Yes**

**No**

14. If yes, name of the reared insects and technique used for domestication?

15. Are insects marketed?

**Yes**

**No**

16. If yes,

a. Name of the marketing place: ___________________________________
 b. Name of the insects sold at the markets: ___________________________

c. Rate of the edible insects: ______________________________________

17. What are the insect and insect products sold by you? **(For insect farmers/sellers)**

a. Name of the insect/ insect product: _______________________________

b. Rate of selling: ______________________________________________

c. Quantity of insects sold per day: _________________________________

d. Income on weekly basis (in rupees):

**0- 5,000/- 5,001-10,000/- above 10,000/-**

18. Are insects used for cultural practices?

**Yes**

**No**

19. If yes, name of the insects used and how?

20. Are there any insect based folk stories in your community?

**Yes**

**No**

21. If yes, narrate the story.

22. Do you prefer insects over meat?

**Yes**

**No**

a. If yes, name of the insects preferred and why?

b. If no, why do you believe meat is better?

23. Are edible insects used in animal feed?

**Yes**

**No**

24. If yes,

a. Name of the insect used: _____________________________________

b. Mode of preparation: ________________________________________

25. Are edible insects used as baits?

**Yes**

**No**

26. If yes,

a. Name of the insect used: ______________________________________

b. Mode of use: _______________________________________________

27. Do you manage insect pest for your cultivated crops?

**Yes**

**No**

28. If yes,

a. Chemical or non-chemical: _____________________________________

b. Regular or irregular use: _______________________________________

29. Are there any measures initiated to conserve edible insects?

**Yes**

**No**

30. If yes,
 a. Name of the community/organization: ____________________________

b. Methods used for conservation: _________________________________

31. Give your feedback on availability of edible insects in the region.

**Increased**

**Decreased**

32. Does the younger generation practice entomophagy?

**Yes**

**No**

33. How important are insects according to you?

Date of collecting information ___________________________

Place of collecting information __________________________

Name of enumerator __________________________________
