# Supplementary material for: Entomotherapy: a study of medicinal insects of seven ethnic groups in Nagaland, North-East India
Source: J Ethnobiol Ethnomed. 2021 Mar 22;17:17. doi: 10.1186/s13002-021-00444-1 (PMC7986042; doi:10.1186/s13002-021-00444-1)
Supplement: Supplementary file 1 — Additional file 1: Supplementary file 1. Demographic patterns of informants in the study area. Supplementary file 2. QUESTIONNAIRE FORMAT. [file 13002_2021_444_MOESM1_ESM.zip › SUPPLEMENTARY MATERIAL S1.pdf]

Supplementary file 2: QUESTIONNAIRE FORMAT

Name of the informant: ____

Age:

☐ **25-34** ☐ **35-44** ☐ **45-54**

☐ **55-64** ☐ **65-74** ☐ **75-84**

☐ **85-94** ☐ **95+**

Sex:

☐ **Male**

☐ **Female**

Community: ________________ Local dialect: _________________________

Qualification:

☐ **Below high school**

☐ **Above high school**

Occupation:

☐ **Government servant**

☐ **Farmer**

☐ **Unemployed**

Informant:

☐ **Key informant**

☐ **General informant**

Contact No.: _________________ Strength of family: _____________________

Place of interview:

a. Name of place/ village: ____

b. Location/ District: ____

c. Other details: ____

d. Signature: ____

1. Do you eat insects?

☐ **Yes**

☐ **No**

2. If yes, can you name the edible insects consumed by you?

Sl. no	Common name	Local name	Seasonal availability	Edible stage	Mode of consumption	Therapeutic value	Myths/ Folklore
1							
2							
3							
4							
5							

3. If no, can you name the edible insects consumed by others?

   a. Local name of the insects: ____________________________________

   b. Name of the tribe consuming the insects: ________________________

   c. Edible parts: ____

   d. Edible stage: ____

   e. Habitat of the insects: **Terrestrial/Aquatic/Burrowing**

   f. Time of collection: ____

   g. Seasonal availability: _______________________________________

   h. Mode of collection: ____

   i. Mode of preparation and consumption: __________________________

4. Are there any indigenous methods for capturing insects?

   ☐ **Yes**

   ☐ **No**

5. If yes, can you name them and explain in details?

6. Are chemicals used for capturing insects?

   ☐ **Yes**

   ☐ **No**

7. If yes,

   a. Name of the chemical: _________________________

   b. Name of the insect captured: ____________________

8. Do you preserve the edible insects for future consumption?

   ☐ **Yes**

   ☐ **No**

9. If yes, then how are they preserved?

☐ **Fermented**

☐ **Smoked**

☐ **Sundried**

☐ **As Pickle**

Others: ____

10. Is entomophagy (insect consumption) popular in the region?

☐ **Yes**

☐ **No**

11. If yes, what are the most popular edible insects? Name them.

12. How often do you take/consume insects?

☐ **Everyday**

☐ **Once in a week**

☐ **Several days in a week**

☐ **Never**

13. Are insects reared?

☐ **Yes**

☐ **No**

14. If yes, name of the reared insects and technique used for domestication?

15. Are insects marketed?

☐ **Yes**

☐ **No**

16. If yes,

a. Name of the marketing place: __________________________________

b. Name of the insects sold at the markets: __________________________

c. Rate of the edible insects: _____________________________________

17. What are the insect and insect products sold by you? **(For insect farmers/sellers)**

a. Name of the insect/ insect product: ______________________________

b. Rate of selling: ____

c. Quantity of insects sold per day: ________________________________

d. Income on weekly basis (in rupees):

☐ **0- 5,000/-** ☐ **5,001-10,000/-** ☐ **above 10,000/-**

18. Are insects used for cultural practices?

☐ **Yes**

☐ **No**

19. If yes, name of the insects used and how?

20. Are there any insect based folk stories in your community?

☐ **Yes**

☐ **No**

21. If yes, narrate the story.

22. Do you prefer insects over meat?

☐ **Yes**

☐ **No**

a. If yes, name of the insects preferred and why?

b. If no, why do you believe meat is better?

23. Are edible insects used in animal feed?

☐ **Yes**

☐ **No**

24. If yes,

a. Name of the insect used: ____________________________________

b. Mode of preparation: ____

25. Are edible insects used as baits?

☐ **Yes**

☐ **No**

26. If yes,

a. Name of the insect used: _____________________________________

b. Mode of use: ____

27. Do you manage insect pest for your cultivated crops?

☐ **Yes**

☐ **No**

28. If yes,

a. Chemical or non-chemical: ______________________________

b. Regular or irregular use: ______________________________

29. Are there any measures initiated to conserve edible insects?

☐ **Yes**

☐ **No**

30. If yes,

a. Name of the community/organization: ______________________

b. Methods used for conservation: __________________________

31. Give your feedback on availability of edible insects in the region.

☐ **Increased**

☐ **Decreased**

32. Does the younger generation practice entomophagy?

☐ **Yes**

☐ **No**

33. How important are insects according to you?

Date of collecting information ______________________

Place of collecting information _____________________

Name of enumerator __________________________
